# Sex Differences in Older Adults' Immune Responses to Seasonal Influenza Vaccination

**DOI:** 10.3389/fimmu.2019.00180

**Published:** 2019-02-27

**Authors:** Emily A. Voigt, Inna G. Ovsyannikova, Richard B. Kennedy, Diane E. Grill, Krista M. Goergen, Daniel J. Schaid, Gregory A. Poland

**Affiliations:** ^1^Mayo Clinic Vaccine Research Group, Mayo Clinic, Rochester, MN, United States; ^2^Division of Biostatistics, Mayo Clinic, Rochester, MN, United States

**Keywords:** sexual dimorphism, influenza vaccine, influenza, elderly, vaccinomics, systems biology, sex differences, immunity

## Abstract

**Background:** Sex differences in immune responses to influenza vaccine may impact efficacy across populations.

**Methods:** In a cohort of 138 older adults (50–74 years old), we measured influenza A/H1N1 antibody titers, B-cell ELISPOT response, PBMC transcriptomics, and PBMC cell compositions at 0, 3, and 28 days post-immunization with the 2010/11 seasonal inactivated influenza vaccine.

**Results:** We identified higher B-cell ELISPOT responses in females than males. Potential mechanisms for sex effects were identified in four gene clusters related to T, NK, and B cells. Mediation analysis indicated that sex-dependent expression in T and NK cell genes can be partially attributed to higher CD4+ T cell and lower NK cell fractions in females. We identified strong sex effects in 135 B cell genes whose expression correlates with ELISPOT measures, and found that cell subset differences did not explain the effect of sex on these genes' expression. Post-vaccination expression of these genes, however, mediated 41% of the sex effect on ELISPOT responses.

**Conclusions:** These results improve our understanding of sexual dimorphism in immunity and influenza vaccine response.

## Introduction

Since 2010, seasonal influenza A is believed to have killed between 12,000 and 79,000 people, has resulted in 140,000–960,000 excess hospitalizations annually in the United States (1), and incurs annual costs of nearly $90 billion (2). During the 2017–2018 influenza season, seasonal influenza caused nearly 80,000 deaths in the United States alone (3). Males and females exhibit differences in immune responses to many viral vaccines, with females generally developing significantly higher levels of humoral immunity than males, including in response to the seasonal influenza vaccine (4–16). Seasonal influenza infection in males also tends to result in worse outcomes than infection in females (17–20), an effect most pronounced in the elderly (17). As seasonal influenza vaccine efficacy is sub-optimal, and particularly variable in the older adults most likely to be seriously affected by disease (21–27), sex-based differences in vaccine response further enhance inter-individual differences in influenza protection across populations and exacerbate this major public health issue.

Sex-based differences in vaccine-induced immune responses can be observed in pre-pubertal children, throughout the reproductive years, and have been demonstrated to persist after menopause (4–13, 28–30). However, even with mounting evidence of significant differences in immune responses to vaccines based on sex, most vaccine studies do not analyze immune response outcome data by sex (31, 32). In particular, little is known about sex effects in cellular (non-antibody) immune responses to influenza vaccine, or potential mechanisms for sex-differential immune responses to vaccines. A single published study examined sex differences in gene expression after influenza vaccination, proposing the involvement of a testosterone-regulated lipid metabolism pathway differentially expressed in males and females for which normalized gene expression correlated inversely with vaccine response (adjusted odds ratio of 0.39 for males compared to 2.25 for females) (14). Additional studies are necessary to confirm and expand these results and improve our understanding of how an individual's sex affects immunity.

## Methods

The study population and laboratory methods described herein are similar or identical to those published in our previous studies (33–38).

### Recruitment

The original study cohort comprised 159 generally healthy older adults of Caucasian descent, ages 50–74, 62% female, who received the 2010/11 seasonal trivalent inactivated influenza vaccine (TIV; Fluarix by GlaxoSmithKline, lot AFLUA524AA; containing the A/California/7/2009 NYMC X-191 [H1N1], A/Victoria/210/2009 NYMC X-187 [H3N2; an A/Perth/16/2009-like virus], and B/Brisbane/60/2008 viral strains), which was given by standard protocol into the deltoid muscle using a 16-gauge, 1-inch needle (38, 39). Blood (100 ml each) samples were taken immediately before vaccination as well as 3 and 28 days after vaccination by professional phlebotomists in Mayo Clinic's Clinical Trials Unit (38). Recruitment was performed at Mayo Clinic, Rochester, MN. Full immune datasets (successful measurement of HAI and VNA antibody titers, B cell ELISPOT response counts, transcriptomic data, and medical records of biological sex) were successfully obtained from 138 subjects (of whom 66% were female), and these data were used for all analyses, as described previously (40). Of these 138 subjects, immune cell phenotypes were successfully obtained from 135 subjects using flow cytometry, and these data were used for plots and statistical tests incorporating immune cell subsets. A brief table with cohort characteristics is included in Supplementary Table 1. Immune outcomes and some transcriptomic data from this cohort have been previously published (36–38, 41–44); the sex-differential statistical analyses and results reported herein have not been previously published.

### Ethics Statement

Written informed consent was obtained from each study participant, and the Mayo Clinic Institutional Review Board approved the study. Influenza virus was propagated in embryonated chicken eggs as described (35). Mayo Clinic's Institutional Animal Care and Use Committee (IACUC) policy does not require review of research on unhatched embryonated eggs destroyed before hatching, in accordance with the Office of Laboratory Animal Welfare and the National Institutes of Health policy.

### Source of Biological Materials

Madin-Darby Canine Kidney (MDCK) cells were obtained from the American Type Culture Collection.

### Isolation of Peripheral Blood Mononuclear Cells (PBMCs)

PBMCs were isolated from subject-derived whole blood samples, as described previously using BD Vacutainer® CPT™ cell preparation tubes with sodium citrate (33). The cells were cryopreserved prior to sequencing and use in assays (33–35).

### Flow Cytometric Measurement of Immune Cell Composition

Subjects' immune cell subsets were quantified using flow cytometry (45). Briefly, frozen subject PBMCs drawn at baseline were thawed and stained using fluorochrome-conjugated antibodies (Abs) from BD Biosciences (San Jose, CA). B cells, NK cells, NKT cells, monocytes, and dendritic cells were identified using the following panel of Abs: CD11c-V450; CD3V500; CD86-fluorescein isothiocyanate (FITC); CD56-phycoerythrin (PE); CD123-allophycocyanin (APC); CD20-Peridinin chlorophyll protein complex (PerCP)-Cy5.5; HLA-DR-Alexfluor 700; CD16-PE-Cy7; and CD14-APC-Cy7. CD4+, and CD8+ T cells were distinguished using the following panel of Abs: CD3-Brilliant Violet 421; CD4-AlexaFluor 700; CD25-Brilliant Violet 605; and CD127-AlexaFluor 647. Following staining, cells were fixed with 4% paraformaldehyde, and data were collected using a BD LSR II Flow Cytometer with FACSDIVA software (BD Biosciences). These antibody panels were chosen by the Human Immunology Project Consortium to allow for comparison of immunophenotyping data across studies (46). Cell population gating was performed using FLOWJO (FlowJo, LLC, Ashland, OR, United States). Debris and doublets were removed using FSC and SSC parameters. Cell subsets were identified based on the surface staining patterns indicated in Supplementary Table 2. Data represent the mean of technical quadruplicates from each subject.

### Growth of Influenza Virus

Influenza A/California/7/2009/H1N1-like virus was provided by the Centers for Disease Control and Prevention (Atlanta, GA, United States). As previously described, the virus was propagated in embryonated chicken eggs and harvested from the allantoic fluid 48 h post-inoculation. Fifty-percent tissue culture infectious dose (TCID_50_) measurements of virus stocks were determined by infection of MDCK cells with serial dilutions of the virus, and dilution wells with successful virus replication were determined by addition of red blood cells 5 days after infection and observation of hemagglutination as per standard protocols (47–49).

### Measurement of Serum Antibody Responses to Influenza Vaccine

The methods for assessing both the hemagglutination-inhibition influenza A/H1N1 antibody (HAI) titers and virus-neutralizing influenza A/H1N1antibody (VNA) titers for these subjects have been published (34–37). Ab titers were measured from sera collected in BD Vacutainer Serum tubes at each timepoint pre- and post-vaccination using standard protocols. Serum influenza A/H1N1-specific neutralizing antibody titers were measured by a cell-based microneutralization assay (assay CV = 4.7%) at each timepoint with influenza A/H1N1 virus stimulation (200 plaque-forming units per 5 μl), as previously described (37). Median values from triplicate technical replicates were used for data analysis.

### Measurement of Humoral Adaptive Immune Responses to Influenza Vaccine

Influenza-specific B cells were measured by influenza A/H1N1-specific B-cell ELISPOT after *in vitro* stimulation of subject PBMCs with vaccine-strain A/H1N1 influenza virus, as previously described (35, 36, 40). Briefly, ELISPOT analyses using the MabTech Human IgG ELISpot^PLUS^ Kit (Mabtech, Inc.; Cincinnati, OH) (35) were used to quantify the influenza A/H1N1-specific B cells (memory-like IgG B cells) in subjects' PBMCs. ELISPOT plates were coated with a 1:50 dilution of influenza A/H1N1 virus stock (50,000 TCID_50_/well). The median of four technical replicates was used for data analysis.

### mRNA-seq

Methods for transcriptomic sequencing were published in our previous transcriptomics studies (43, 50). Briefly, we extracted total RNA from each cryopreserved subject PBMC sample using RNAprotect reagent and RNeasy Plus mini kits reagent (Qiagen; Valencia, CA, United States). Poly-A RNA was isolated using magnetic purification, and Mayo Clinic's Gene Sequencing Facility created cDNA libraries using the mRNA-Seq 8 Sample Prep Kit (Illumina; San Diego, CA). An Illumina HiSeq 2000 was used to perform single-end read sequencing. The human genome build 37.1 was used to align sequencing reads using TopHat (1.3.3) and Bowtie (0.12.7).

The resulting mRNA-sequencing gene-count data underwent a strict quality-control and normalization procedure, as described by Ovsyannikova et al. (43). Briefly, Conditional Quantile Normalization (51) was used to normalize gene counts; 14,197 genes were determined to have at least 32 counts at one of our three timepoints (Day 0, 3, or 28) and were used in our subsequent analyses.

### Statistical Analysis of Sex Differences in Immune Cell Compositions and Single Gene Expression Levels

Wilcoxon rank-sum tests were used to test for differences between males and females in CD4+ T cell percentage and NK cells percentage and single gene-level gene expression. Spearman's correlation was used to test for correlation between NK cell percent and CD4+ T cell percent in each subject as well as for correlation between single gene expression levels and Day 28 B cell ELISPOT responses. Story and Tibshrani's method (43) for genome-wide studies was used to calculate q-values for these gene level results.

### Weighted Gene Coexpression Network Analysis (WGCNA)

Because the expression of genes can be highly correlated, we chose to focus on clusters (modules) of highly correlated genes and use summary information of each cluster as a measure of gene expression of multiple correlated genes. This was accomplished by Weighted Gene Coexpression Network Analysis (WGCNA), as previously described (40). Using the Day 28 normalized gene expression data, we created data-driven gene clusters using WGCNA via creation of a co-expression similarity matrix followed by hierarchical clustering techniques (52–54). The first principal component of the gene expression levels within a gene cluster represent the cluster's eigengene and served to represent the entire cluster's gene expression activity. Pearson's correlation was used to correlate immune phenotypes with each cluster's eigengene to identify the gene clusters related to vaccine responses.

### Gene Enrichment Analyses

Enrichment analysis for genes involved in known pathways or functions was previously performed on the gene clusters using the RITAN (55) package and published Blood Translation Modules (BTMs) (56), as described previously (40).

### Mediation Analyses and Calculation of Sex Effect in Antibody Titers, ELISPOT Result, and Gene Cluster Expression Levels

Causal mediation analysis was conducted using the “mediation” package in R version 3.4.1 (57, 58). The eigengenes from each WGCNA cluster were used as the outcome variables, and we analyzed whether the fraction of CD4 + T cells or NK cells in PBMCs mediate the relationship between sex and the gene expression eigengenes. Linear models were used for analyses, and all continuous variables were scaled to have the mean equal to zero and standard deviation equal to one. The non-parametric bootstrap method was used with 1,000 simulations to estimate variance of the model parameters and calculate the 95% confidence intervals. Results of mediation analyses include the average direct effect (ADE), average causal mediated effect (ACME), and total effect (TE). The TEs from these models were used as an estimate of the effect of sex on the gene expression clusters at each time-point (Day 0, 3, and 28). False discovery q-values were calculated using the Benjamini and Hochberg method (59). Gene clusters were reported as potentially sex-dependent if they had a sex-difference *p* < 0.01 and *q* < 0.1 at a minimum of two of the three time points measured.

We analyzed whether cluster gene expression (summarized by eigengenes) mediates the relationship between sex and B-cell ELISPOT responses and whether cell subset percentage mediates the relationship between sex and gene expression in sex-dependent gene clusters using the same framework. The TEs from these models are used to estimate the effect of sex on ELISPOT responses and antibody responses or cell subset % on gene cluster expression. The ACMEs calculated by mediation analysis are reported as estimates of the percent of the total sex effect mediated by cluster gene expression or cell subset %. False discovery q-values were calculated for the mediation analysis results using the Benjamini and Hochberg method (59).

## Results

### Males and Females Display Differences in Immune Outcomes After Vaccination

**Figure 1:**
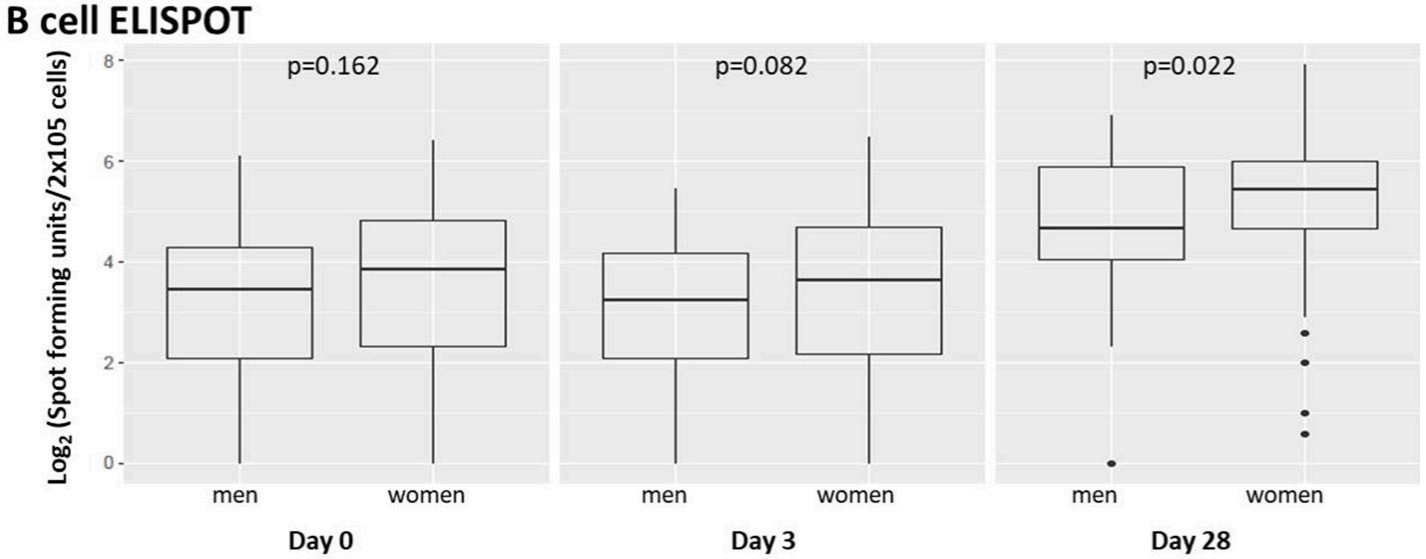
Memory B-cell ELISPOT responses to influenza virus differ by subject sex. Memory B cells capable of responding to vaccine strain influenza A/H1N1 were quantified using B-cell ELISPOT. Dot plots indicating individuals' responses are available in Supplementary Figure 1.

To assess the effect of subject sex on humoral immunity after influenza vaccination, we compared males' vs. females' influenza A/H1N1-specific B-cell ELISPOT responses in subject PBMCs harvested from each subject and cryopreserved (35) immediately prior to vaccination, 3 days after vaccination, and 28 days after vaccination (Figure 1).

We found suggestive differences between male and female influenza-reactive B-cell populations 3 days after vaccination and significant differences (*p* = 0.022) 28 days post-vaccination.

**Figure 2:**
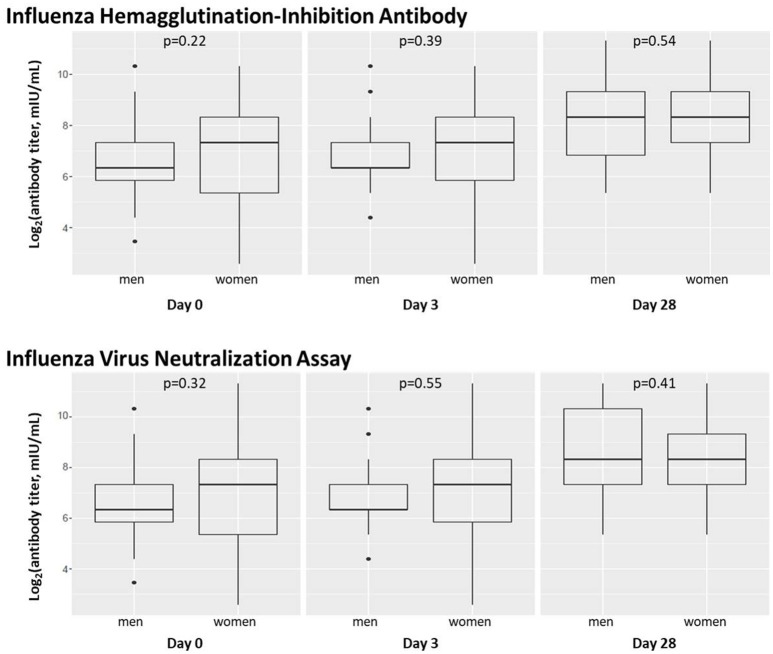
Subject sex does not significantly affect influenza A/H1N1 antibody titers. Box plots are shown for levels of influenza A/H1N1-reactive antibody in subject sera before and after vaccination using hemagglutination-inhibition and virus neutralization assays. Dot plots indicating individuals' responses are available in Supplementary Figure 2.

We also assessed the effect of subject sex on influenza antibody titers immediately prior to vaccination, 3 days after vaccination, and 28 days after vaccination as measured by HAI and VNA assays (Figure 2).

Females had higher median antibody titers at baseline and Day 3, but these differences were not statistically significant (*p* > 0.1). Median antibody titers in males and females were equivalent (1:320) at Day 28. HAI and VNA antibody measures were highly correlated, as previously described (36, 44, 45).

### Sex Differences Are Found in Gene Expression Signatures Related to NK Cell, T cell, and B cell Activity

We also examined gene expression data to identify gene expression clusters whose expression levels are affected by subject sex. Such information may provide clues to the biological source(s) of sex differences in immune responses to influenza vaccination. In a previous study using Weighted Gene Coexpression Network Analysis (WGCNA) clustering of transcriptomic data, we identified 15 non-overlapping clusters of genes that shared similar expression patterns across the study cohort (40). In the current study, we tested the expression levels of each of these gene clusters for sex dependence; four gene clusters demonstrated significant sex dependence (*p* < 0.01 and FDR *q* < 0.1) during at least one of the three timepoints (Table 1). A full list of all quantified genes, their cluster membership, and median expression levels in males vs. females can be found in Supplementary Table 3. Genes located on the X chromosome were distributed evenly across the 15 gene clusters and comprised no more than 5% of any individual gene cluster, and thus are not responsible for observed sex dependence in overall gene cluster expression.

**Table 1:** Sex differences are found in PBMC expression of gene clusters related to immune cell activity, including CD4+ T cell, B cell, and NK cell activity.

**WGCNA gene cluster**	**# genes in cluster**	**Cluster summary:**	Blood translation modules (BTMs) most strongly represented^*^ in this cluster:	**Cluster correlated with:**	Expression sex effect size^**^(***p*****-value)**		
					**Day 0**	**Day 3**	**Day 28**
1	135	B cell activity	Plasma cells and B cells, immunoglobulins (M156.0) Enriched in B cells (I), (II), (III), (VI) (M47.0, M47.1, M69) B cell surface signature (S2)	B-cell ELISPOT responses	0.36 (0.024)	**0.39 (0.026)**	**0.52 (0.004)**
2	629	T cell activity	T cell activation (I) (M7.0) Enriched in T cells (I) (M7.1) T cell activation (III) (M7.4) T cell differentiation (M14)	PBMC cytokine secretion, B-cell ELISPOT	**0.49 (0.004)**	**0.40 (0.022)**	0.29 (0.116)
3	225	NK and T cell activity	Enriched in NK cells (I) (M7.2) Enriched in NK cells (II) (M61.0) Enriched in T cells (I) (M7.0)	HAI/VNA antibody responses	−0.08 (0.678)	**−0.47 (0.014)**	**−0.50 (0.002)**
4	96	NK cell activity	NK cells surface signature (S1) Enriched in NK cells (I) (M7.2) Enriched in NK cells (II) (M61.0)	HAI/VNA antibody responses	−0.27 (0.146)	**−0.51 (0.008)**	**−0.50 (0.006)**

*WGCNA gene clusters whose expression is significantly different between males and females are listed, along with the results of enrichment analysis for these gene clusters (40) and any immune outcomes that correlate significantly with the gene cluster's expression. Pink shading indicates significantly higher cluster gene expression in females, while blue shading indicates higher gene expression in males. Gene clusters were included that had a sex-difference p < 0.01 and q < 0.1 at a minimum of two of the three time points measured. Ninety-five percent confidence intervals for sex-effect sizes can be found in Supplementary Table 4*.

* *BTMs most strongly represented in the cluster as reported in Voigt et al. (40); that work's Greenyellow, Black, Purple, and Salmon gene clusters correspond to WGCNA gene clusters. #1–4 reported here*.

** *Effect size presented is calculated using the first principal component (FPC) of gene expression in each gene cluster and represents the fraction of inter-individual variance in cluster gene expression FPC that can be attributed to subject sex. Higher expression in females relative to males is indicated by a positive effect size (red shading); higher expression in males relative to females is indicated by a negative effect size (blue shading)*.

*Values in bold indicate q < 0.1 in FDR multiple testing correction*.

Females demonstrated significantly higher expression of genes in a cluster of B cell-related genes (#1) than males at Day 28 (*p* = 0.004)—an effect that was also present during earlier timepoints (*p* = 0.024 at Day 0, *p* = 0.026 at Day 3). Females also demonstrated higher expression of T cell genes (cluster #2) than males at baseline and early in vaccine response (*p* = 0.004 at baseline; *p* = 0.022 at Day 3). In gene clusters #3 and #4 enriched for NK and cytotoxic T cell genes, we found higher gene expression in males than females during the response phase (cluster #3, *p* = 0.014 [Day 3] and 0.002 [Day 28]; cluster #4, *p* = 0.008 [Day 3], and 0.006 [Day 28]). Statistical significance of eight of these nine *p*-values was confirmed after FDR calculation of q-values for multiple testing correction; only the Day 1 B-cell gene cluster (#1) sex differences did not meet a *q*-value threshold of < 0.1.

### Sex Differences in B-cell ELISPOT Levels Are Mediated by Sex-Related Differences in Expression of B-cell Related Genes

**Figure 3:**
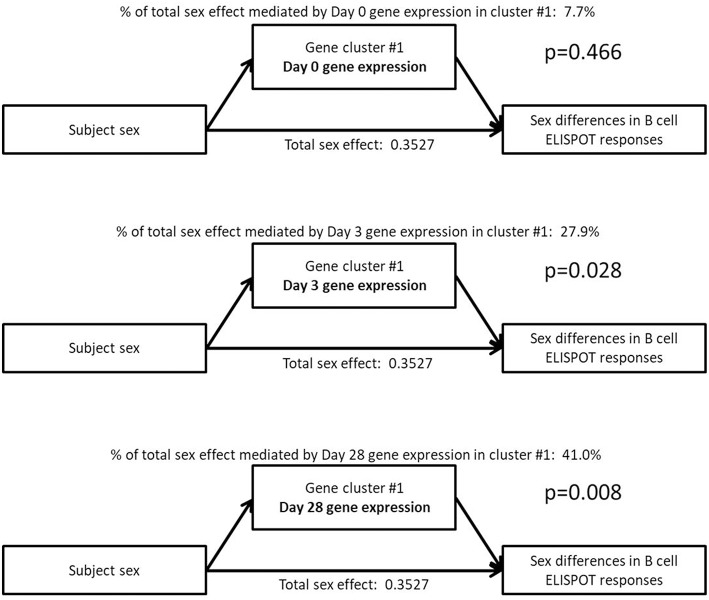
Mediation analysis indicates that the sex effect on B-cell ELISPOT responses is mediated by Day 3 and Day 28 gene expression in a small gene cluster of 135 genes highly enriched for B cell-related genes. The total sex effect represents the fraction of the standard deviation in ELISPOT response that can be explained by sex.

As a step toward identifying the biological source of the sex differences observed in B-cell ELISPOT results, we explored whether the sex differences observed in the expression levels of these gene clusters would statistically explain the sex differences observed in B-cell ELISPOT results. To do this, we utilized statistical mediation analysis to examine whether the gene expression of each cluster *mediates* the effect of subject sex on B-cell ELISPOT responses in our subjects (Figure 3).

Sex differences in Day 28 B-cell ELISPOT results were determined to be mediated by sex-dependent gene expression of a single gene cluster after vaccination (cluster #1: B cell genes, mediating effect *p*-values of 0.028 and 0.008 for Day 3, and Day 28 cluster expression, respectively). This gene cluster represents 135 B cell genes whose postvaccination expression levels we previously showed to correlate strongly with Day 28 B-cell ELISPOT responses (Day 3 correlation 0.28, *p* = 0.001; Day 28 correlation 0.3, *p* = 3 × 10^−4^) (40). Sex-dependent gene expression in gene cluster #1 at 28 days postvaccination explains 41% of the effect of sex on Day 28 B-cell ELISPOT responses, according to our mediation analysis. Gene expression in other gene clusters did not significantly mediate the effect of sex on B-cell ELISPOT responses (*p* > 0.05).

### Males and Females Have Different Immune Cell Compositions That May Affect Immune Responses

**Figure 4:**
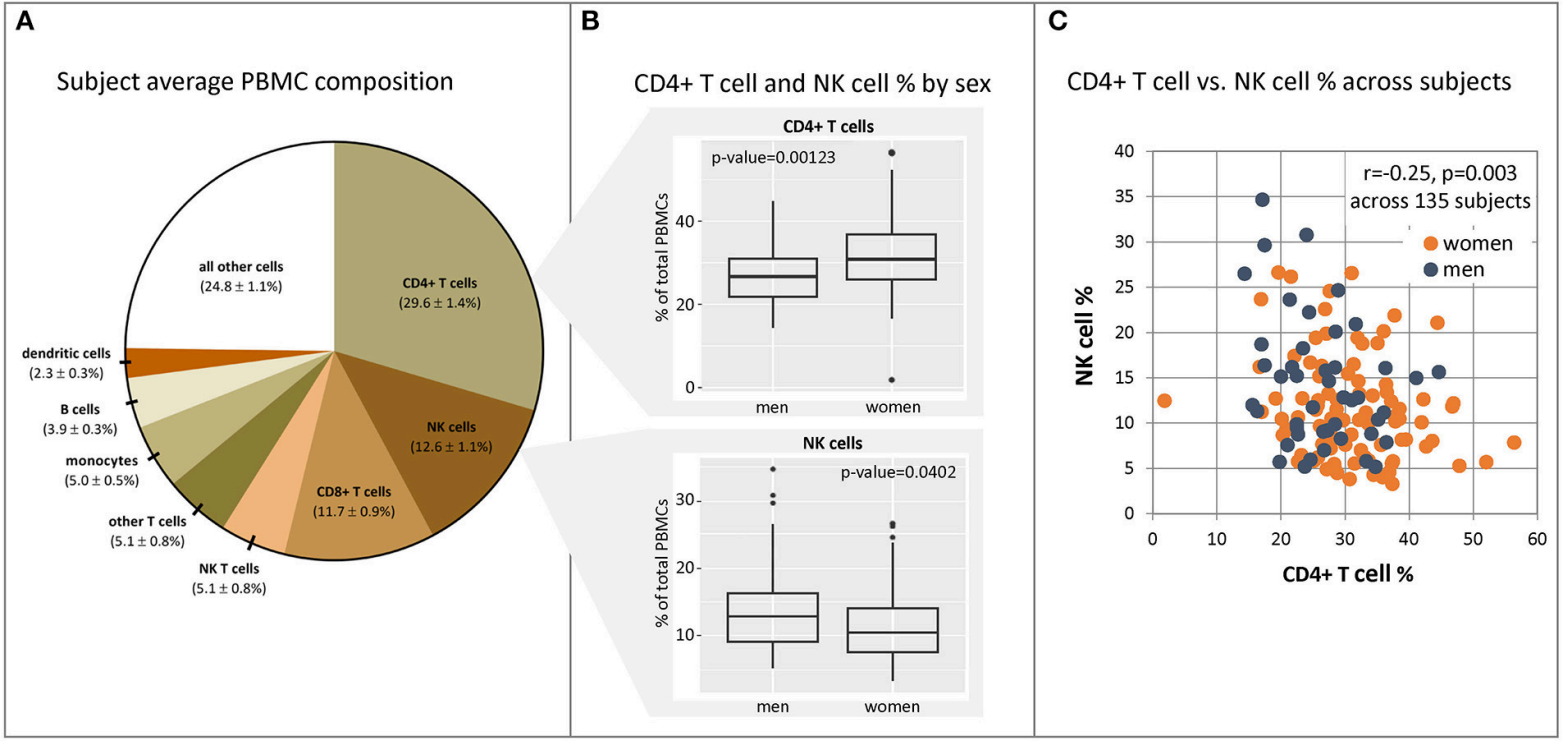
CD4+ and NK cell fractions in PBMCs differ between males and females. PBMC samples were harvested from our cohort subjects immediately prior to vaccination, and populations of immune cell subsets were quantified in each subject sample using fluorescent antibody staining for unique cellular markers and flow cytometry. **(A)** mean PBMC composition and 95% confidence intervals across 135 subjects. **(B)** Significantly different fractions of CD4+ T cells and NK cells were found in males (*n* = 45) vs. females (*n* = 90). Dot plots indicating individuals' responses are available in Supplementary Figure 3. **(C)** CD4+ T cell and NK cell fractions in each subject show weak correlation with one another. Source data may be found in Supplementary Table 5.

One variable that could account for these observed sex differences in gene expression is sex differences in immune cell subset composition and quantity. To explore differences in resting immune states between males and females, we examined baseline differences in immune cell composition between males and females. PBMCs from the 135 subjects with sufficient remaining cryopreserved samples were stained with a panel of fluorescent antibodies to cell markers designed to differentiate and quantify subsets of immune cells via flow cytometry. Specifically, populations of T cells (with CD4+ and CD8+ subsets quantified), B cells, NK cells, NK T cells, monocytes, and dendritic cells were quantified relative to one another (Figure 4).

The average composition of subject PBMCs prior to vaccination is displayed in Figure 4A. Analysis demonstrated a significantly higher fraction of CD4+ T cells in females relative to males (Wilcoxon Rank Sum test, 31.2 vs. 27.1%, *p* = 0.001), and correspondingly higher fractions of NK cells in males relative to females (13.5 vs. 10.5%, *p* = 0.04) (panel B). Other cell populations (B cells, monocytes, dendritic cells, NK T cells, and CD8+ T cells) were not found to be significantly different between males and females. An effect of age was observed for baseline CD8+ T cell and NK cell levels; there was a modest decrease in CD8+ T cell fractions as age increased (Spearman rank correlation = −0.18, *p* = 0.034) as well as an increase in NK cell fractions (Spearman rank correlation = 0.21, *p* = 0.011). No significant effect of age was seen on any other cell type across the age range (50–74 years, *p* > 0.2 for all cell subsets, Spearman rank correlation). CD4+ T cell and NK cell fractions in each subject demonstrated a weak inverse correlation with one another, suggesting that these populations may or may not be regulated in part by the same factors (Panel C).

### Sex Differences in Subject PBMC Immune Cell Compositions Mediate Sex Differences in NK and T cell Gene Cluster Expression Levels, but do not Affect B cell Gene Clusters or ELISPOT Measures

**Figure 5:**
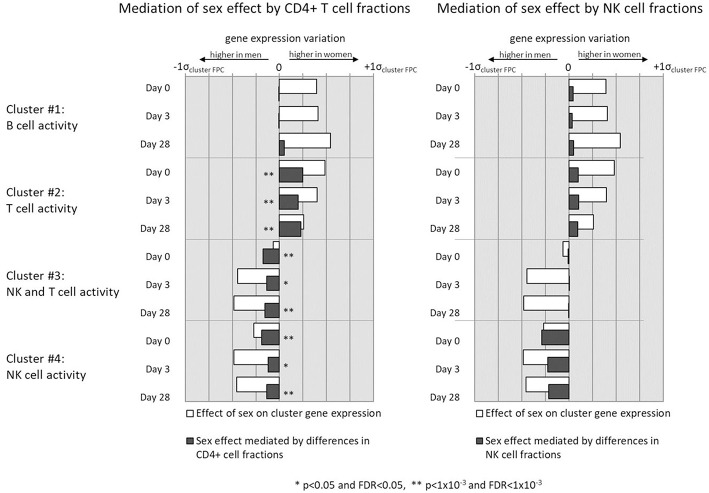
Mediation analysis indicates whether sex-related differences in gene expression of select gene clusters is mediated by observed sex differences in CD4+ cell **(left column)** and NK cell **(right column)** fractions of subject PBMCs.

We observed higher levels of gene expression in females than males in gene clusters that corresponded to T helper cell activity (cluster #2), which also corresponded with the higher fractions of CD4+ T cells observed in females. Similarly, the lower levels of NK cells in females' overall PBMC compositions corresponded with lower gene expression in NK cell-related gene clusters (clusters #3 and #4). To test if the differences in cell subset composition could statistically explain the observed sex differences in gene cluster expression levels, we conducted mediation analyses to determine whether cell subset composition *mediates* the effect of subject sex on gene expression levels in these gene clusters (Figure 5).

Sex differences in CD4+ cell counts were found to partially mediate sex differences in NK and T cell gene clusters #2, #3, and #4 to a highly significant degree. NK cell differences also mediate the effect of sex on gene clusters #2 and #3, which are involved in NK and T cell activity, but to a less statistically significant degree that did not retain significance after multiple testing correction (0.01 < *p* < 0.05, *q* > 0.1). In no gene cluster did differences in immune cell quantities mediate the entirety of the observed sex effect on gene expression. Neither NK cell nor CD4+ T cell sex differences mediated the sex differences found in B cell gene activity (cluster #1), likely reflecting other biological mechanisms as primary contributors to sex effects on B cell gene expression. Finally, neither B cell, NK cell, nor CD4+ T cell fractions between subjects were found to mediate sex differences in ELISPOT response (*p* > 0.1, data not shown).

### B cell Genes That Mediate the Effect of Sex on B-cell ELISPOT Responses Control B cell Proliferation, Differentiation, and BCR Signaling

In order to identify other potential mechanisms for the larger B-cell ELISPOT responses observed in females, we further examined genes in cluster #1 to identify specific genes in this cluster that appear to be expressed in a sex-dependent manner and may therefore be causally mediating these effects. We examined expression levels of individual genes in this cluster and tested for sex-differential expression. The 20 most significantly sex-dependent genes are shown in Table 2 (see Supplementary Table 6 for the complete list of cluster #1 genes).

**Table 2:** Gene cluster #1 genes with significant differences in expression levels between males and females.

**Gene**	Cluster #1 membership^*^	**% higher expression in females relative to males**			***q-*****values, sex differences in expression**			**Gene expression correlation with Day 28 B-cell ELISPOT**			***q*****-values, B-cell ELISPOT correlation**		
		**Day 0**	**Day 3**	**Day 28**	**Day 0**	**Day 3**	**Day 28**	**Day 0**	**Day 3**	**Day 28**	**Day 0**	**Day 3**	**Day 28**
MOXD1	0.66	29.9	44.9	44.0	0.035	0.001	0.000	0.206	0.302	0.404	0.063	0.000	0.000
STAP1	0.83	20.0	17.7	16.7	0.035	0.007	0.001	0.051	0.120	0.165	0.412	0.018	0.001
PAWR	0.77	25.2	24.4	24.0	0.034	0.007	0.001	0.070	0.254	0.235	0.358	0.001	0.000
PTPRK	0.83	19.1	23.3	36.8	0.061	0.007	0.001	0.089	0.233	0.203	0.304	0.001	0.001
CD40	0.54	14.3	16.4	25.6	0.061	0.008	0.001	0.115	0.286	0.270	0.227	0.000	0.000
DPF3	0.75	17.0	13.2	14.6	0.067	0.031	0.001	0.205	0.254	0.256	0.063	0.001	0.000
FCRL5	0.72	6.1	21.2	39.8	0.077	0.031	0.001	0.180	0.250	0.383	0.096	0.001	0.000
CD19	0.93	16.2	25.3	20.2	0.061	0.025	0.002	0.128	0.259	0.329	0.186	0.001	0.000
FAM129C	0.79	14.3	27.9	22.8	0.079	0.008	0.003	−0.047	0.098	0.153	0.417	0.026	0.002
SPIB	0.77	6.2	13.0	26.9	0.077	0.019	0.003	0.132	0.169	0.321	0.180	0.007	0.000
BLNK	0.89	12.3	22.0	16.0	0.061	0.023	0.003	0.087	0.236	0.290	0.312	0.001	0.000
CDCA7L	0.80	7.1	13.8	15.0	0.071	0.007	0.004	−0.045	0.113	0.034	0.421	0.020	0.013
TSPAN13	0.66	11.9	30.7	15.9	0.078	0.007	0.004	−0.074	0.122	0.087	0.349	0.017	0.006
KHDRBS2	0.74	21.3	16.4	25.1	0.077	0.008	0.004	0.009	0.167	0.203	0.533	0.007	0.001
NXPH4	0.62	38.9	30.6	33.1	0.061	0.011	0.004	0.213	0.298	0.316	0.057	0.000	0.000
RALGPS2	0.94	19.3	14.4	17.4	0.061	0.025	0.004	0.100	.290	0.304	0.263	0.000	0.000
CD22	0.94	15.6	20.7	13.4	0.071	0.025	0.004	0.067	0.255	0.279	0.358	0.001	0.000
FCRL2	0.78	25.2	34.1	33.1	0.079	0.031	0.004	0.123	0.220	0.303	0.202	0.002	0.000
ADD2	0.46	9.6	11.6	18.3	0.079	0.043	0.004	0.151	0.263	0.243	0.138	0.001	0.000
TCL6	0.70	9.4	12.3	47.6	0.094	0.044	0.004	−0.012	0.078	0.093	0.530	0.036	0.006

* *Cluster #1 membership is the correlation between the median expression of the individual gene with the cluster eigengene*.

*Red indicates the degree of higher gene expression in females relative to males. Green indicates the degree of correlation between gene expression and subject ELISPOT reponses. The 20 most sex-dependent genes are displayed; for the full table of all 135 Cluster #1 genes, see Supplementary Table 6*.

We found 116 genes that demonstrated sex-differential expression at a minimum of one timepoint after multiple testing correction (*q* < 0.05). The expression of most of these genes also correlated significantly with Day 28 B-cell ELISPOT measures across the subjects and represent potential links between sex-dependent gene expression and B-cell ELISPOT measures.

These genes fall into two distinct categories. Approximately half of these genes have known direct functions in B cell signaling, proliferation, and differentiation (e.g., *CD40, FCRL5, CD19, BLNK, EBF1*). That these genes are more highly expressed in females relative to males indicates the heightened activity of females' B cells after vaccination relative to those of males. The remaining sex-dependent genes are largely transcription factors and are also likely to be active in B cell responses to vaccination, with links to cell fate decision making (e.g., *SOX5, DPF3*), cell proliferation/differentiation (e.g., *SPIB*), and cell signaling (e.g., *MOXD1, PTPRK, SPIB, KHDRBS2*). The sex-differential expression of these genes after vaccination reinforces that while B cell fractions are similar in males and females, intrinsic B cell signaling, proliferation, and differentiation activities are significantly higher in females. Our evidence points to this collection of genes as likely to be critically involved in the sex-differential immune outcomes observed after influenza vaccination.

## Discussion

### Summary of Findings

We found higher levels of influenza A-specific memory B cells in females relative to males after seasonal influenza vaccination. Previous studies investigating sex differences in seasonal influenza vaccine responses have largely examined antibody titers, reaching no clear consensus (8, 9, 44, 60, 61). Additionally, the ability to identify subtle sex differences in antibody responses is limited by the variability and log-based nature of antibody titer assays. Our study is the first to identify sex differences using the sensitive influenza-specific memory B cell ELISPOT assay as a key measure of long-term vaccine-induced immune memory.

Transcriptional sex differences in vaccine responses were observed in four gene clusters highly enriched for NK cell, T cell, or B cell genes. The strongest sex effect was noted for a small gene cluster of 135 B cell genes (*p* = 0.004). The post-vaccination expression of genes in this cluster alone was found to statistically mediate a substantial fraction of the effect of subject sex on B-cell ELISPOT responses (mediation *p*-value 0.008). No other gene clusters were found to mediate the sex effects on ELISPOT, regardless of the sex dependence of their expression levels.

**Figure 6:**
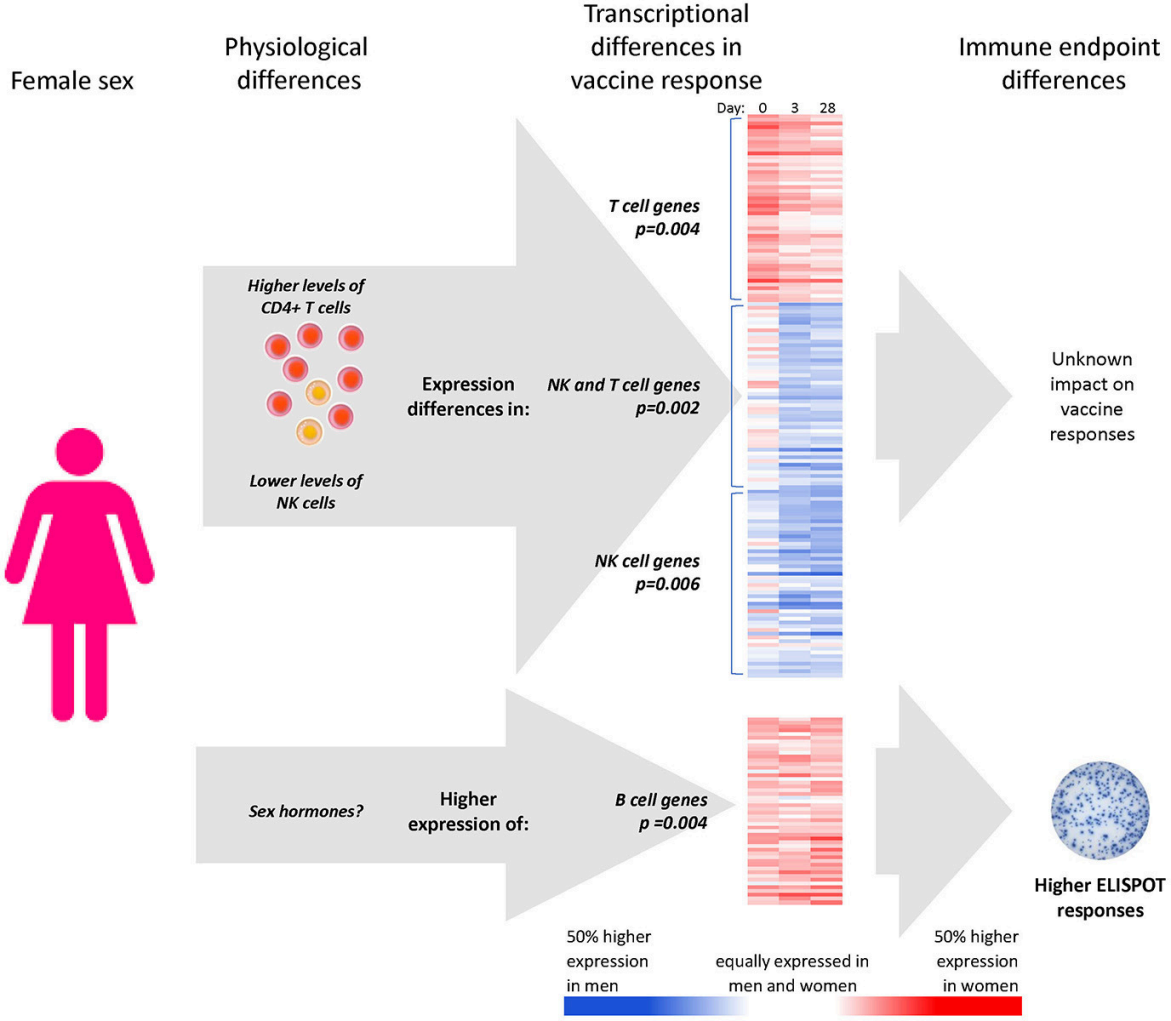
Apparent mechanisms of sex effects on influenza vaccine responses. CD4+ T cell and NK cell numbers mediate part of the effect of subject sex on the expression levels of gene clusters involved in NK cell and T cell genes, with unknown impact on immune outcomes. Higher expression of 135 B cell genes in females was not found to relate to sex differences in immune cells; however, it was determined that these B cell genes were, however, statistical mediators of the higher B-cell ELISPOT responses to seasonal influenza vaccine found in females relative to males. ^*^Gene expression heatmaps present the sex-differential expression of the 50 most representative genes of each sex-dependent gene cluster (i.e., most highly correlated with the cluster eigengene).

Immune cell composition has previously been shown to affect responses to vaccination (36, 62). Here, we demonstrate that males and females have different PBMC fractions of CD4+ T cells and NK cells, and that these differences mediate a significant part of the sex-differential expression levels in T cell and NK cell gene clusters after influenza vaccination (Figure 6). However, the sex-differential gene expression of B cells that ultimately leads to higher B-cell ELISPOT responses in females appears to be independent of CD4+ and NK cell sex differences.

### Sex Differences in CD4+ T cells

Influenza-specific memory T cells play an important role in the protection of individuals against influenza disease (63–65). While serum antibody measures are often used as a correlate of protection, influenza antibody titers are limited as measures of vaccine efficacy, particularly in older individuals (63–65). Populations of influenza-reactive memory CD4+ T cells, on the other hand, have been repeatedly demonstrated to correlate well with protection in both younger and elderly adults (64, 65). These CD4+ T cell responses are induced in the weeks after influenza vaccination and may be better predictors of long-term immunity in healthy adults than other measures of immunity (66). Higher net levels of CD4+ T cells in women relative to men may, therefore, significantly affect an individual's ability to respond effectively to influenza vaccines. Indeed, female mice have been shown to have greater levels of CD4+ T cell responses than males when exposed to the 2009 H1N1 strain of influenza virus (15).

Ours is not the first study to have identified larger fractions of total CD4+ T cells in females' PBMCs relative to males'. In large studies of healthy individuals spanning a broad age range, Amadori et al. and Wikby et al. also found no effect of sex on CD8+ T cell populations but higher CD4+ T cell counts and higher CD4/CD8 ratios in females (67, 68). Interestingly, while CD4/CD8 ratios have been previously found to remain relatively constant in individuals over multiple-year time frames (67), inverted CD4/CD8 ratios have also been shown to increase with age over decades-long time scales and have been associated with a higher mortality risk in individuals over the age of 60 (68, 69).

These sex-differential CD4/CD8 cell ratios appear to be at least partly sex hormone-mediated, as indicated in studies of androgen-deficient men, and women after surgical menopause (70–74). Indeed, hormone therapy was demonstrated to reverse the hormone-deficiency effects on CD4/CD8 ratios in both these situations; however, the timeframe necessary for hormone treatment to achieve such effects is unclear. Natural monthly menstrual changes in sex hormones were found to have no apparent effect on CD4/CD8 T cell ratios (67); thus, hormone differences may need to exist for extended periods of time—from many months to years—for effects on CD4+ T cell populations to be observed (75).

In addition to higher numbers of CD4+ T cells in females relative to males, the ability of these cells to respond to vaccination appears to differ substantially between sexes. For example, CD4+ T cells express estrogen receptors (76), and estrogen has been demonstrated to impact T cell development, mature T-cell function, and the establishment of T cell memory (77–81). The methylation and transcription patterns of males' and females' T cells are, as a result, quite distinct (82, 83).

In older individuals, this sex effect on T cell activity may exacerbate concomitant age effects on immunity. Studies have previously demonstrated that naïve CD4 functions decrease dramatically with age and contribute to reduced humoral responses to vaccination (63, 78, 84–87). Our analysis showed higher age-corrected gene expression of many genes relative to naïve T cell functions in female subjects relative to males. Examples include baseline expression of *CD28* (*p* = 0.004) and the genome-organizer *SATB1* (*p* = 0.003), which play critical roles in activating T cell genes and shaping the T cell pool (88, 89). We hypothesize that higher levels of CD4+ T cells, combined with potentially higher activity as suggested by others (90), may help older females retain net T_h_ cell function over time and maintain the ability to successfully respond to vaccination.

### Sex Differences in NK Cells and Inflammation

Others have also observed higher levels of NK cells in males relative to females. NK-cell numbers also appear to be regulated by sex hormones (75). The role of NK cells in vaccine response has particular implications in older populations. Proinflammatory cytokines, such as IL-6, IL-1β, and TNFα, are produced at elevated levels in elderly persons (91). This phenomenon, termed “inflammaging,” may be due to chronic stimulation of innate immunity, which has been demonstrated to negatively impact immune responses to vaccination (92–94), perhaps by masking the stimuli induced by vaccines (95). Larger fractions of NK cells in male PBMCs and greater activity of proinflammatory NK cell-related genes suggest that older males may experience a higher degree of inflammaging than older females. This may add to the difficulty in developing protective vaccine-induced immune responses in older males.

### Sex Differences in B cell Responses to Vaccination

We found that total B cell fractions did not differ significantly between men and women; however, the numbers of influenza-specific B cells after vaccination as measured by ELISPOT were significantly higher in women than men. These results suggest that sex differences do not originate in differences in total numbers of B cells, but in a heightened ability of women to respond to vaccination and generate long-lasting influenza-reactive memory B cells. Supporting this hypothesis is the fact that we observed clear sex-related differences in B cell gene expression after vaccination. For example, *CD40* gene expression in PBMCs was significantly higher in females than males across the timepoints in our study (*p* < 0.01) (Supplementary Table 6), implying that female antigen-presenting cells may be inherently better poised to proliferate and differentiate in response to vaccine-activated CD4+ T cells (96, 97).

Evidence in the literature corroborates the existence of sex-based differences in B cell immunity. For example, a large microarray study (*n* = 5,241) of normal adults in the absence of any immune stimulation found strong female-biased expression of genes involved in immune system processes, including regulation of immunity, response to cytokines, and regulation and activation of leukocyte differentiation (98). In mice exposed to the 2009 strain of H1N1 influenza, antibody from females demonstrated both greater antibody responses and increased antibody avidity and specificity than in males (15). While the mechanism behind differences in B cell gene expression is not well-defined, evidence exists for both sex hormone-mediated and sex hormone-independent components. The existence of sex differences in infants' responses to vaccination indicates that non-hormonal sex differences in immunity exist. However, estrogens and other sex hormones also play clear roles in immunity; in the aforementioned microarray study (98), the gene transcription differences between males and females appear to be at least partially linked to estrogen levels—largest in females using hormonal contraceptives and smaller, though still present, in postmenopausal females. Similarly, *in vivo* estrogen levels were found to relate to lymphocyte homeostasis in another study (99). These results confirm that sex differences in B cell responses to immunization are likely a combination of hormone-mediated and hormone-independent effects.

### Sex Hormone Effects on Immunity

Estrogen, testosterone, and other sex hormones have been observed to clearly impact B cell activity; these effects may be direct, indirect, or both. B cells express, for example, ERα/β estrogen receptors, allowing estrogen-related compounds to directly modulate lymphocyte function. *In vitro* treatment of both male and female human PBMCs with 17-beta estradiol was demonstrated decades ago to significantly enhance B cell differentiation (100, 101). Estrogen-related compounds have been demonstrated to directly increase B cells' expression of anti-apoptotic molecules critical for B cell activity and development, while androgen treatment decreases expression of such molecules (102–104). Such hormone-modulated gene expression appears to affect diverse B cell functions from lymphopoesis to BCR signaling, B cell expansion, and B cell maturation (103–106), as reviewed by Sakiani et al. (107). Direct binding of estrogen receptors has several effects on activated B cells, including increased antibody production, somatic hypermutation, class-switch recombination, and on the development and persistence of B cell memory (108–113).

Testosterone, while not demonstrated to directly influence B cells *in vitro* to the extent of estrogen (100, 101, 114), has also been implicated in sex-based differential vaccine responses. In a systems biology analysis of data from 91 young and old individuals by Furman et al. (14), high testosterone levels in men were associated with poor virus-neutralizing response. This study further identified a cluster of 35 lipid metabolism genes known to be testosterone-regulated whose expression related to poor virus-neutralizing activity in men. We successfully quantified 22 of these 35 lipid metabolism-related genes in our current study, yet we did not find any consistent sex differences in these genes' expression to replicate this result despite a significantly larger sample size. As Furman et al. noted, the effect of these 35 genes' expression was most notable in males with high testosterone levels. As our cohort comprises solely older individuals with likely lower testosterone levels due to age, the effects of high testosterone males may simply no longer be observable; however, we did not measure testosterone levels in our study.

The presence of estrogen and testosterone may also impact B cells indirectly via their effects on other immune cells. Though at levels lower than in B cells, estrogen receptors are present on a large array of immune cells, including T cells, NK cells, dendritic cells, and monocytes (76, 115). It is well-established that sex hormones regulate innate immune functions of monocytes, dendritic cells, and macrophages, which affect NFκB and TLR signaling, interferon secretion, and proinflammatory cytokine production (98, 116–122). Evidence exists for the involvement of monocytes and/or T cells in estrogen-based augmentation of antibody responses in PBMCs both *in vitro* and *in vivo* (123, 124).

While the precise cellular mechanisms behind the effects of sex hormones on immunity are still being fully defined, the impact of estrogen and other sex hormones on humoral and cellular immune responses is not a new concept. It has been suggested for decades that estrogen enhances humoral and possibly cellular immune responses and is involved in the pathogenesis of various autoimmune diseases found predominantly in females, such as systemic lupus erythematosus (114, 125, 126).

### Hormone-Independent Sex Effects on Immunity

While sex-differential immune responses to vaccines have been noted in infants and pre-pubertal children (4, 127–130) in whom large hormonal differences do not exist, non-hormonal sex differences have not been well-studied or understood. The existence of different immune-regulating genes and miRNAs on the X and Y chromosomes has been suggested as a mechanism for genetic and epigenetic, rather than hormonal, sex-related differences in immune responses (131–133). While our study demonstrated that X-chromosome genes comprised < 5% of any gene cluster, transcriptional differences in a few key regulatory genes could significantly affect the immune response system and contribute to the observed sex differences. Our study provides a list of 135 B cell genes that may be important mediators of this effect. In a follow-up study, it would be interesting to examine the influence of X-linked genes on the expression of these mediators of sex-differential ELISPOT responses. This information may lead to further refinement of our understanding of how biological sex influences immunity.

Some have additionally proposed that sex differences in immunity may be mediated by the microbiome, as differences appear to exist between male and female microbiomes, and microbiome compositions in turn affect immune responses (134–140). However, the source of many of these sex differences in microbiome composition is proposed to be sex-hormone mediated (135, 136, 141); thus, it is questionable whether such a mechanism would in fact be hormone-independent or explain infant sex-based differences in immunity. Additional study should be done of sex effects on microbiome compositions and any effects this may have on vaccine responses.

### Study Strengths and Limitations

The strengths of this study include the comprehensive datasets collected from the 138-subject cohort. These data allowed us to examine the sources and potential mechanisms of sex effects on immunity at a level previously impossible in most studies. Additionally, the WGCNA hierarchical gene clustering algorithm, which clustered genes effectively based on expression levels across our 138 subjects (40) may, in fact, be identifying and incorporating sex differences in gene expression into the creation of the gene clusters, despite WGCNA being blind to subject sex. For example, the WGCNA algorithm created multiple gene clusters enriched for T cell genes, one of which (cluster #2 described here) showed strong sex-dependent gene expression while others demonstrated no sex dependence in gene expression and are, as a result, not discussed in detail here. Similarly, the introduction of mediation analysis to this study adds a novel and valuable tool to the field of systems vaccinology by allowing us to statistically connect sex differences in cell subset compositions to sex differences in immune gene expression and immune outcomes.

Our study does, nonetheless, have several limitations. The cohort size of 138 subjects remains relatively small for detecting subtle differences between populations. While our Caucasian cohort allows for excellent male/female comparisons, generalizability to other populations would require further work. Pre-existing immunity is also a potential confounding factor in all influenza vaccine studies. Invariably, some individuals have high levels of pre-existing cellular and/or antibody responses and paradoxically appear not to respond well to vaccination. This may reflect high levels of pre-existing immunity rather than an inability to respond to the vaccine (37). While others have created elaborate algorithms to deal with pre-existing immunity in influenza vaccine transcriptomic studies (142), we previously demonstrated that the WGCNA gene clustering algorithm does not require normalization to baseline expression levels, thus avoiding the need to correct for pre-existing immunity using complex methods (50). Finally, this study was conducted using a seasonal influenza vaccine containing influenza A/H1N1, influenza A/H3N2, and influenza B. While the presented PBMC gene expression data reflect responses to vaccination with all three influenza strains, only influenza A/H1N1-specific neutralizing antibody and memory B-cell ELISPOT assays were conducted due to costs and practical limitations. With additional funding, further studies examining sex differences in ELISPOT and antibody measures of specific responses to the A/H3N2 and influenza B vaccine strains may be performed in the future.

## Summary

We demonstrate in this study that sexually dimorphic components of the immune responses to seasonal influenza vaccination include differences in immune cell populations; expression levels of gene clusters related to T cell, B cell, and NK cell activity; and memory B cell activity as measured by B-cell ELISPOT. Higher memory B cell activity after vaccination was identified in females relative to males—a result correlated to, and statistically mediated by, the expression levels of a small cluster of 135 B cell-related genes. The biological source of these genes' sex-differential expression is unknown, but may be related to human sex hormones and their myriad effects on B cells. This study identifies key genes mediating these effects for future study of the underlying mechanisms. Sex differential responses to seasonal influenza vaccine may impact vaccine efficacy across populations, particularly for older adults with weaker immune responses. Knowledge gained by this and future studies may lead to the development of better vaccines to optimally induce protective immunity in individuals of both sexes, and to the possibility of individualized vaccine practice (143, 144) utilizing adjuvants and other mechanisms.

## Author Contributions

All authors were involved in study conceptualization and reviewed, edited the manuscript. EV and DG prepared the original draft of the manuscript. RK and IO supervised the laboratory work. EV, DG, KG, and DS designed the methodology used for analysis, and KG, DG, and EV performed the formal analysis. GP and IO supervised the research efforts. KG and EV prepared the figures.

### Conflict of Interest Statement

GP is the chair of a Safety Evaluation Committee for novel investigational vaccine trials being conducted by Merck Research Laboratories. GP offers consultative advice on vaccine development to Merck & Co. Inc., Avianax, Adjuvance, Valneva, Medicago, Sanofi Pasteur, GlaxoSmithKline, and Emergent Biosolutions. RK has received funding from Merck Research Laboratories to study waning immunity to mumps vaccine. GP and IO hold patents related to vaccinia and measles peptide research. RK holds a patent related to vaccinia peptide vaccines. These activities have been reviewed by the Mayo Clinic Conflict of Interest Review Board and are conducted in compliance with Mayo Clinic Conflict of Interest policies. This research has been reviewed by the Mayo Clinic Conflict of Interest Review Board and was conducted in compliance with Mayo Clinic Conflict of Interest policies. The remaining authors declare that the research was conducted in the absence of any commercial or financial relationships that could be construed as a potential conflict of interest.
