# Supplementary material for: Sex Differences in Older Adults' Immune Responses to Seasonal Influenza Vaccination
Source: Front Immunol. 2019 Feb 27;10:180. doi: 10.3389/fimmu.2019.00180 (PMC6400991; doi:10.3389/fimmu.2019.00180)
Supplement: Supplementary file 2 [file Table_2.DOCX]

Supplementary Table 2. Cell surface markers used to differentiate immune cell subsets

| **Cell Type** | **Gating Strategy and Surface Markers** |
| --- | --- |
| CD4^+^ T cells | CD3^+^CD4^+^ (also CD127^+^CD25^-^ to exclude regulatory T cells) |
| CD8^+^ T cells | CD3^+^CD4^-^ |
|  | |
| B cells | CD3^-^CD20^+^ |
| NK cells | CD3^-^CD56^+^ |
| NK T cells | CD3^+^CD56^+^ |
| Classical Monocytes | CD3^-^CD20^-^HLA-DR^+^CD14^+^CD16^-^ |
| Non-classical monocytes | CD3^-^CD20^-^HLA-DR^+^CD14^-^CD16^+^ |
| Intermediate monocytes | CD3^-^CD20^-^HLA-DR^+^CD14^+^CD16^+^ |
| Plasmacytoid dendritic cells | CD3^-^CD20^-^HLA-DR^+^CD14^-^CD16^-^CD11c^+^CD123^-^ |
| Myeloid dendritic cells | CD3^-^CD20^-^HLA-DR^+^CD14^-^CD16^-^CD11c^-^CD123^+^ |
