# Supplementary material for: Sex Differences in Older Adults' Immune Responses to Seasonal Influenza Vaccination
Source: Front Immunol. 2019 Feb 27;10:180. doi: 10.3389/fimmu.2019.00180 (PMC6400991; doi:10.3389/fimmu.2019.00180)
Supplement: Supplementary file 7 [file Presentation_1.PPTX]

## Slide 1
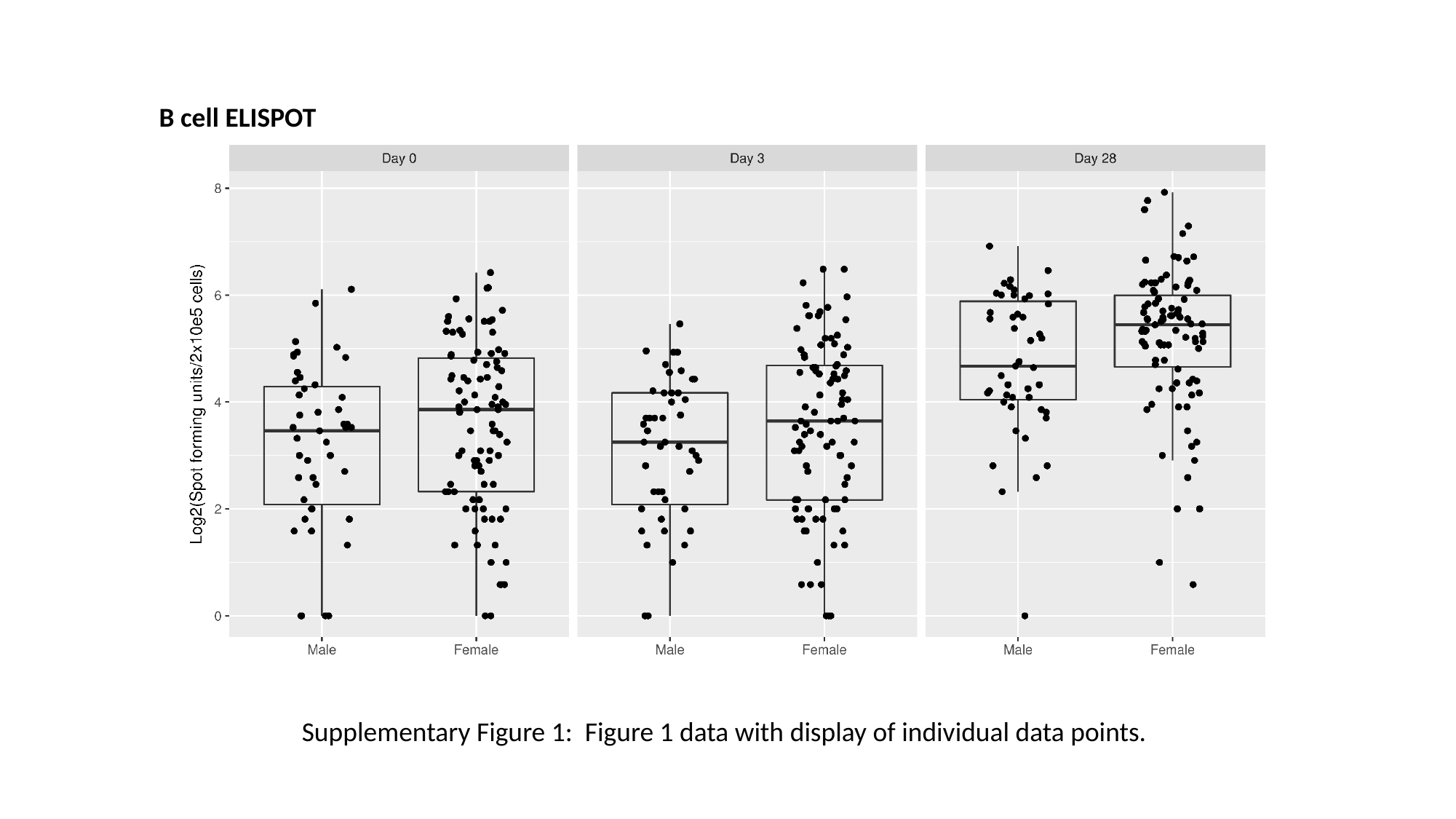

B cell ELISPOT
Supplementary Figure 1: Figure 1 data with display of individual data points.

## Slide 2
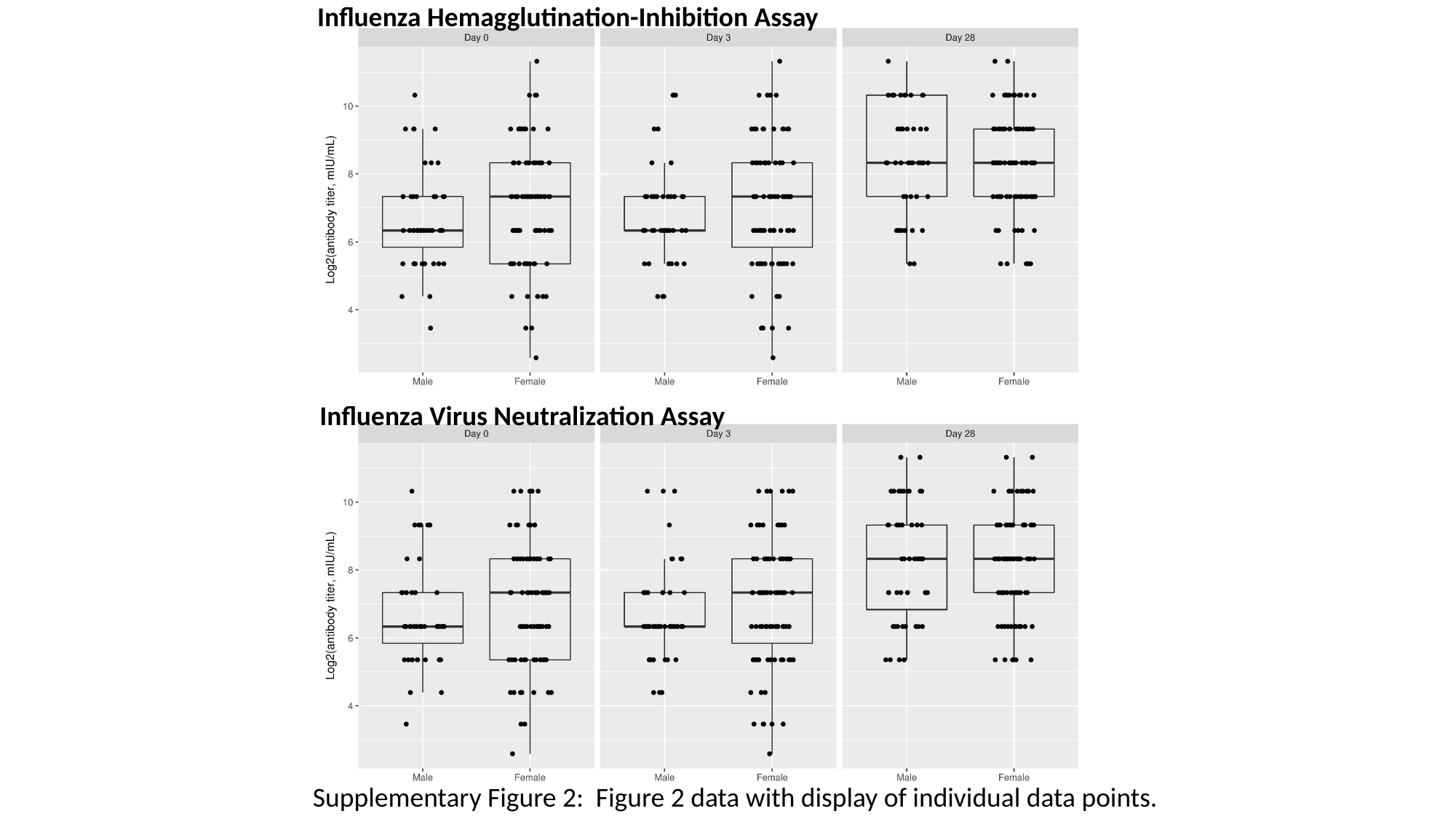

Influenza Hemagglutination-Inhibition Assay
Influenza Virus Neutralization Assay
Supplementary Figure 2: Figure 2 data with display of individual data points.

## Slide 3
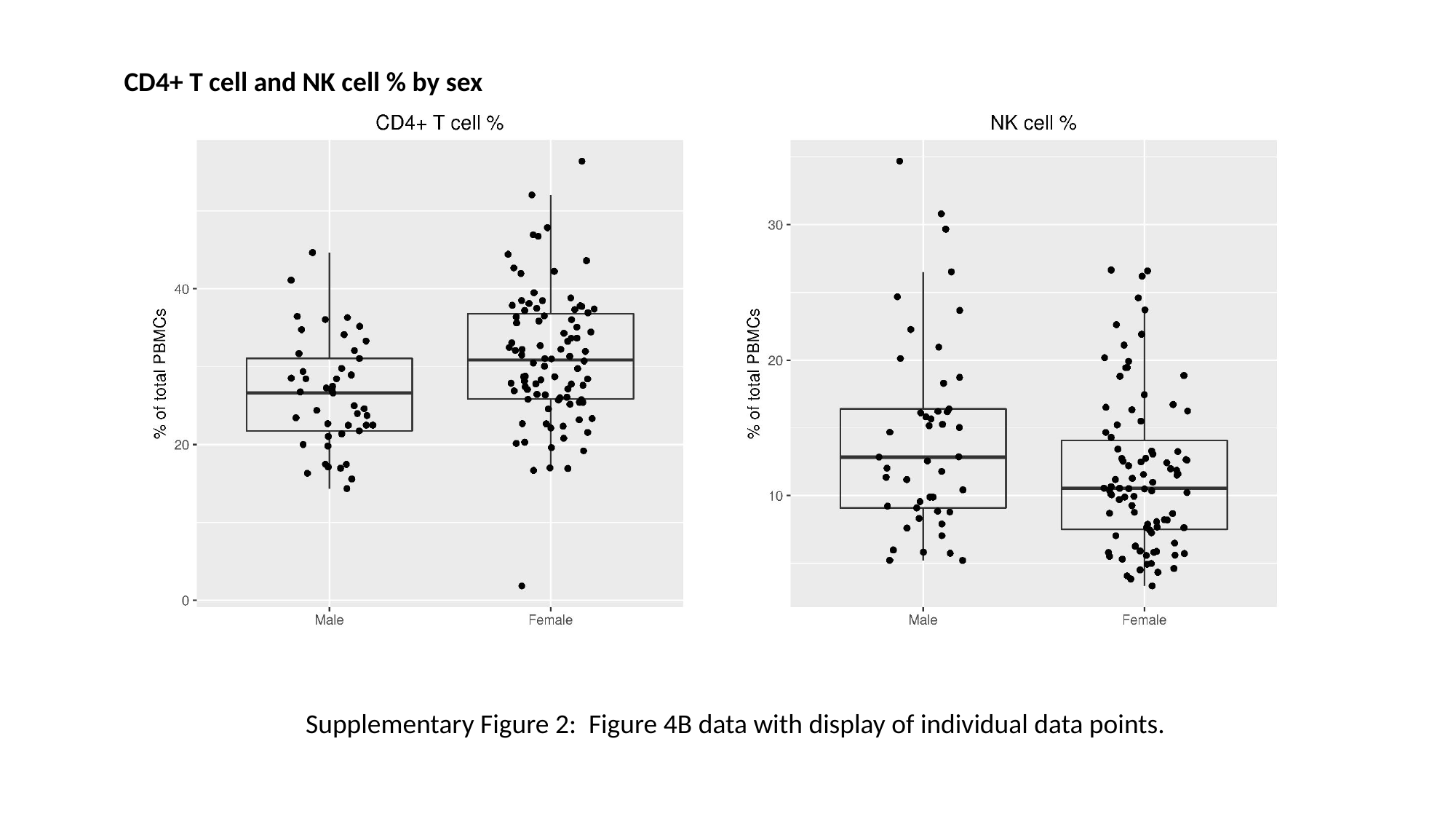

CD4+ T cell and NK cell % by sex
Supplementary Figure 2: Figure 4B data with display of individual data points.
